# A549 cells contain enlarged mitochondria with independently functional clustered mtDNA nucleoids

**DOI:** 10.1371/journal.pone.0249047

**Published:** 2021-03-25

**Authors:** Aleksandrs Nasonovs, Miguel Garcia-Diaz, Daniel F. Bogenhagen

**Affiliations:** Department of Pharmacological Sciences, Stony Brook University, Stony Brook, NY, United States of America; Imagine Institute, FRANCE

## Abstract

Mitochondria are commonly viewed as highly elongated organelles with regularly spaced mtDNA genomes organized as compact nucleoids that generate the local transcripts essential for production of mitochondrial ribosomes and key components of the respiratory chain. In contrast, A549 human lung carcinoma cells frequently contain apparently swollen mitochondria harboring multiple discrete mtDNA nucleoids and RNA processing granules in a contiguous matrix compartment. While this seemingly aberrant mitochondrial morphology is akin to “mito-bulbs” previously described in cells exposed to a variety of genomic stressors, it occurs in A549 cells under typical culture conditions. We provide a detailed confocal and super-resolution microscopic investigation of the incidence of such mito-bulbs in A549 cells. Most mito-bulbs appear stable, engage in active replication and transcription, and maintain respiration but feature an elevated oxidative environment. High concentrations of glucose and/or L-glutamine in growth media promote a greater incidence of mito-bulbs. Furthermore, we demonstrate that treatment of A549 cells with TGFβ suppresses the formation of mito-bulbs while treatment with a specific TGFβ pathway inhibitor substantially increases incidence. This striking heterogeneity of mitochondrial form and function may play an important role in a variety of diseases involving mitochondrial dysfunction.

## Introduction

Mitochondria are semi-autonomous organelles that harbor their own mtDNA genomes encoding 37 genes including tRNAs, rRNAs and 13 mRNAs for electron transport chain components. Typically, healthy mitochondria are elongated tubular structures with a diameter of 400 to 500 nm with a variable degree of branching or reticulation. MtDNA molecules occur as regularly spaced foci called nucleoids containing on average, 1–2 mitochondrial genomes [1,2]. Nucleoids are typically associated with the mitochondrial inner membrane in zones lacking extensive cristae structure [3–5] often in close association with mitochondrial RNA processing granules (MRG) [6].

Mitochondria are remarkably dynamic with cycles of fission and fusion that contribute to quality control [4,7]. One mechanism for this sort of quality control relies on fission to produce small mitochondrial particles that can only rejoin the mitochondrial network if they possess an appropriate membrane potential indicative of healthy respiratory status [8]. Particles that fail quality control are subject to autophagic destruction. In addition to this wholesale autophagy, material may be removed from intact mitochondria by other “piecemeal” methods [7] that help to maintain mitochondrial function.

While autophagy may handle small defective mitochondria effectively, a number of pathological conditions have been reported to result in formation of apparently swollen or bloated mitochondria [9] that may not be culled efficiently by these methods. Understanding the organization of mtDNA genomes in such swollen organelles is a fundamental interest since mitochondrial gene products are essential to support continued synthesis of respiratory complexes. Mitochondrial swelling with nucleoid aggregation has been reported in response to chemical agents such as DNA intercalators [10], uncoupling agents [11] or certain respiratory chain inhibitors [11] as well as by genetic manipulations of TFAM [12], proteins involved in mitochondrial fission and fusion (e.g., Opa I [13], Drp I (DNML1; [14]) and Mfn2 [11]), as well as some mito-ribosomal proteins and putative assembly factors [15]. Recently, mitochondrial swelling with clustered nucleoids has also been reported in Hutchison-Guilford progeria [16].

We recently reported that apparent swelling of mitochondria and mtDNA clustering contributes to development of fibrotic lung disease [17], suggesting that this phenotype may reflect a mitochondrial stress response. During the course of our studies of cultured pulmonary cells, we noted that A549 lung epithelial carcinoma cells frequently contain such bloated mitochondria with three characteristics: a spherical profile, a minimal diameter significantly exceeding 500 nm and containing multiple nucleoids within a single matrix compartment. The mitochondrial morphology is markedly heterogeneous in these cells, with some tubular and some apparently swollen mitochondria. These cells provide an unusual opportunity to study the incidence and consequences of this aberrant mitochondrial morphology in the *absence* of direct genetic manipulation or cytotoxic treatment. The A549 cell line is derived from a non-small-cell lung adenocarcinoma but retains some features of AT2 pulmonary cells [18,19]. These cells have frequently been used as a convenient model for the epithelial-mesenchymal transition (EMT) and its reversion (MET) in response to TGFβ and other influences; possibly including media and culture conditions [20,21].

We used super resolution structured-illumination microscopy (SIM) [22,23] and PALM/STORM microscopy [24] to visualize nucleoids in these bloated organelles. We found that these mitochondria are not transient structures, but persist for at least four hours in culture and can attain a diameter of over 5 μm, particularly when cells are grown in media containing elevated glucose and glutamine concentrations. The inner membrane cristae are poorly developed in these organelles, leaving nucleoids dispersed in an expanded matrix compartment. Such apparently swollen mitochondria exhibit a higher oxidative environment than neighboring mitochondria with normal morphology. Finally, we report that known effectors and blockers of EMT significantly influence the incidence of aberrant mitochondrial morphology in A549 cells [25].

## Results

### Mitochondria in A549 cells frequently exhibit bloated morphology with nucleoids clustered in an expanded matrix space with poorly developed cristae

**Fig 1:**
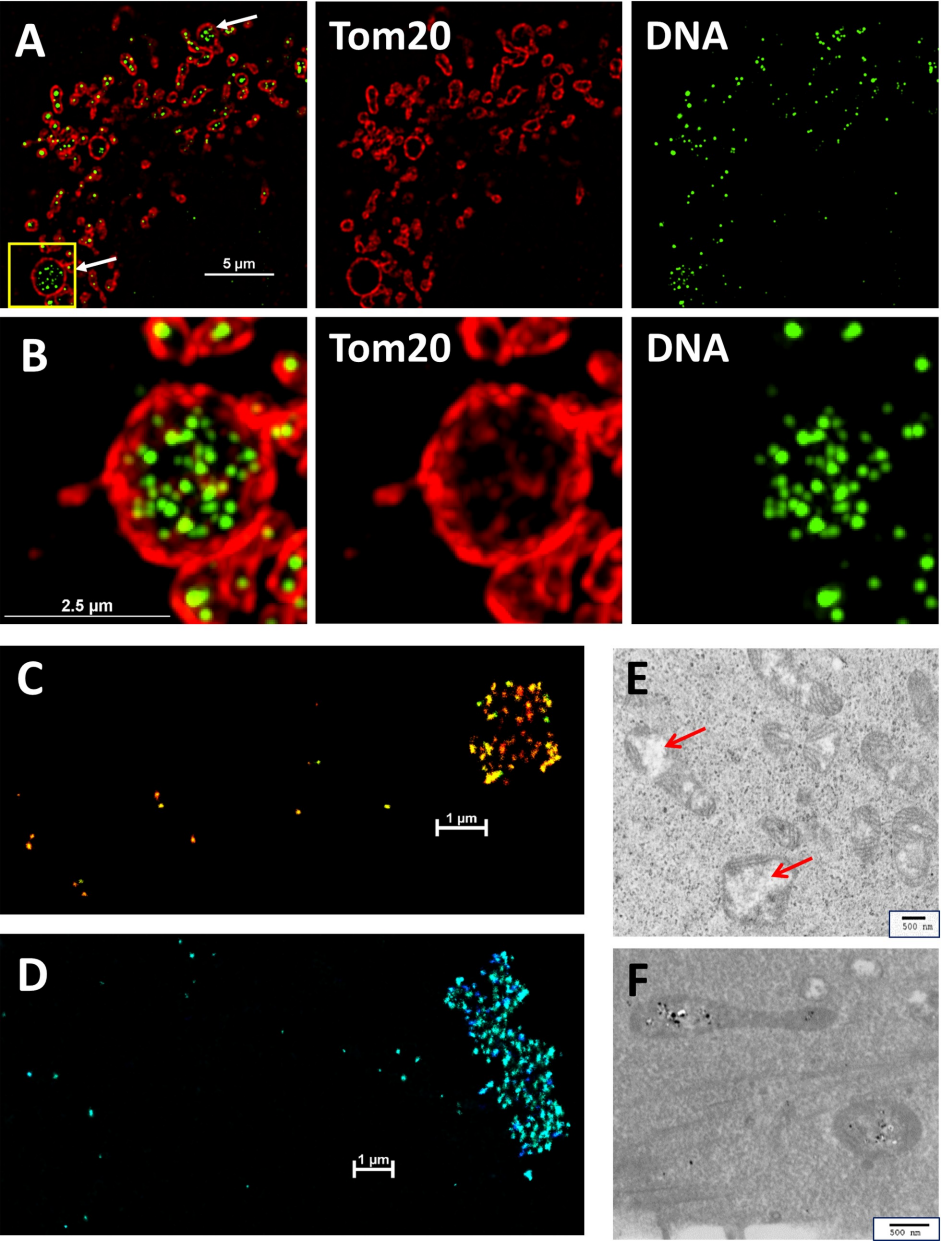
A549 cells grown in DMEM contain bloated mitochondria with multiple clustered mtDNA nucleoids. **A.** SIM image of an A549 cell stained with antibodies against Tom20 (red) and DNA (green) showing nucleoid clusters inside bloated mitochondria (white arrows). Resolved channels are shown on the right. Scale bar is 5 μm. **B.** A 2.875 μm thick section displayed as a 3D maximum projection showing Tom20 and DNA staining from the ROI boxed in yellow in panel A. The expanded right-hand panels show Tom20 and DNA channels resolved independently. NIKON 3D-SIM, z-stack of 23 slices at 125 nm intervals, scale bar is 2.5 μm. **C,D.** 3D-STORM images of two A549 cells stained with anti-DNA antibodies showing both individual nucleoids and clustered nucleoids. Images are pseudo-colored to show depth in the section. Scale bars 1 μm. **E.** TEM depicts bloated mitochondria highlighting prominent electron lucent areas devoid of cristae (red arrows). Scale bar is 500 nm. **F.** TEM image showing bloated mitochondria with mtDNA decorated with anti-DNA Nano-gold labeling within electron lucent regions. Scale bar is 500 nm.

During the course of experiments to investigate mitochondrial morphology in pulmonary epithelial cells, we noted a striking degree of clustering of nucleoids within bloated mitochondria in A549 cells under normal culture conditions. Unlike classical tubular mitochondria, some mitochondria in A549 cells occur as oblate spheroids with diameters approaching 3–5 μm in some cases, with aggregated distinct mtDNA nucleoids (Figs 1A, 1B and S1). Such nucleoid clustering is in direct contrast to the individual, periodically spaced, distribution of individual nucleoids typically found in tubular mitochondria (S2B Fig).

Notably, these nucleoid clusters do not merge into single enlarged aggregates of mtDNA. 3D-STORM imaging of anti-DNA labeled A549 cells grown in DMEM revealed nucleoids as either isolated structures or in large clusters (Fig 1C and 1D). Measurements of nucleoid sizes revealed no significant difference in the sizes of individual isolated or clustered nucleoids (Table 1A). EM images show that bloated mitochondria possess large electron lucent spaces (Fig 1E). Electron lucent spaces are also noted to occur in regions of swelling, and stain with anti-DNA antibodies (Fig 1F). Similar enlarged organelles have sometimes been referred to as “mito-bulbs” [14]. We employ this term with the understanding that swollen mitochondria with clustered nucleoids can be generated in diverse ways and that the properties of mito-bulbs may vary depending on the cell type or circumstances leading to their appearance. We refer to these organelles interchangeably as “swollen or bloated mitochondria” or “mito-bulbs.” This usage is not intended to convey a specific mechanism for the apparent swelling, which may or may not result from ion and water transport across the mitochondrial inner membrane as described by Javadov et al. [26]. We have observed mito-bulbs in A549 strains received from colleagues and validated by PCR as well as those received directly from ATCC. An informal review of several dozen publications using A549 cells showed that about half of the studies used DMEM while a comparable number used RPMI. In our hands, A549 cells cultured in RPMI appear to feature predominantly a tubular, interconnected, mitochondrial network indicative of normal “healthy” mitochondria while A549 cells cultured in DMEM commonly have a more fragmented network featuring shorter mitochondria with a more spherical profile (S1A and S1B Fig). Although, we observe mito-bulbs in both media, they appear more frequently when A549 cells are adapted and cultured in DMEM, as opposed to RPMI, despite the fact that cells grow at very similar rates in both media (S1C Fig). SIM analysis revealed nearly three times as many clusters of three or more nucleoids, contained inside roughly spherical organelles, for A549 cells grown in DMEM as opposed to those grown in RPMI (Table 1B). To provide additional statistical evidence of the greater incidence of mito-bulbs in DMEM grown cells, we analyzed numerous images using a watershed algorithm quantifying the number of bloated organelles that exceed a series of threshold sizes (S1D and S1E Fig). Our data reveals that the incidence of mito-bulbs increases with time spent on the culture dish after seeding in either medium, but is consistently higher when cells are cultured in DMEM.

**Table 1:** Incidence of mtDNA nucleoid clustering in A549 cells.

**A. Nucleoid Size and Shape**		
	**Single**	**Clustered**
**Number**	22	24
**Semi-diameter**	75.3+19.6 nm	85.1+18.1 nm
**Ellipticity**	1.48+.24	1.46+.36
**B. Incidence of clustered nucleoids in cross sections**		
	**DMEM**	**RPMI**
**No Cells**	27	18
**Total nucleoids**	3777	2955
**Clustered nucleoids**	763	187
**% clustered**	20.2	6.3

**A.** A549 cells stained with anti-DNA antibodies were imaged using 3D STORM to measure minimum and maximum diameters in the XY plane to permit calculation of the mean values and standard deviation of the semi-diameter and ellipticity.

**B.** Single slice SIM images of the indicated numbers of cells grown for multiple generations in either DMEM or RPMI medium and stained with anti-DNA and anti-Tom20 antibodies were analyzed with object counting tools in the Nikon AR software to quantify the total numbers of nucleoids in cross-sections in the indicated numbers of cells. Nucleoids in organelle sections with at least three nucleoids in a single matrix space were quantified manually.

**Fig 2:**
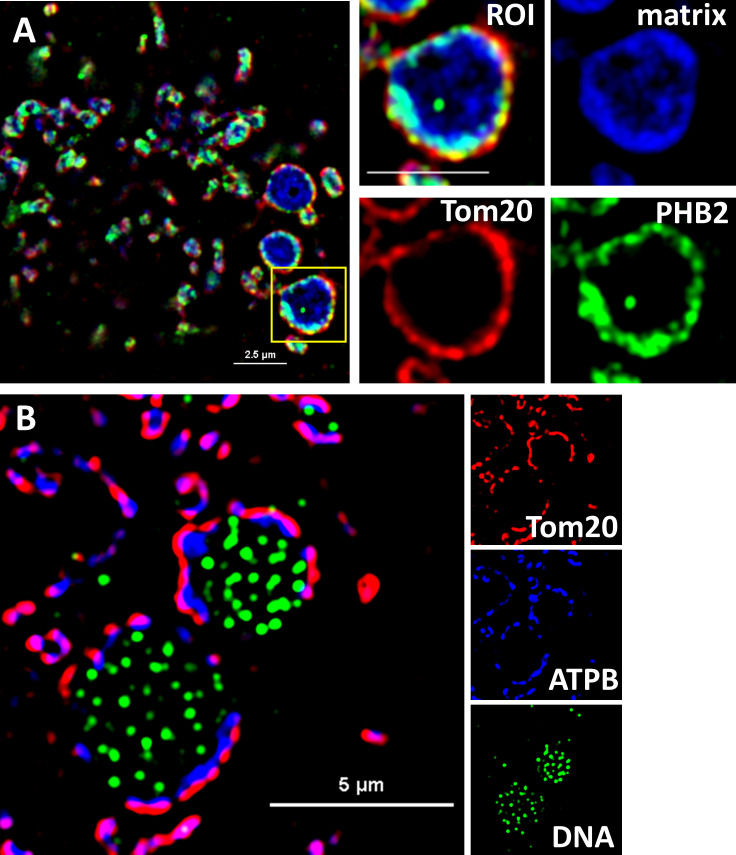
Mito-bulbs in A549 cells exhibit poor inner membrane organization in matrix regions containing clustered mtDNA nucleoids. **A.** SIM image depicts mito-bulbs in a DMEM-adapted A549 cell expressing MLS-EGFP (blue) enclosed by markers for the outer membrane (Tom20, red) and inner membrane (PHB2, green). The yellow boxed ROI is expanded and channels resolved in panels on the right. Scale bar is 2.5 μm. **B.** SIM image of a DMEM-adapted A549 cell stained with antibodies against outer membrane Tom20 (red), inner membrane marker ATPB (blue) and DNA (green), shown as individual channels on the right. Scale bar is 5 μm.

The large electron-lucent spaces evident in TEM images of bloated mitochondria appear to lack extensive cristae (Fig 1E). In typical mitochondria the inner membrane (IM) is composed of two distinct domains, the inner boundary membrane (IBM), closely apposed to the outer membrane (OM), and the cristae membrane formed by extensive in-foldings [27] so that the IM is estimated to have several times the surface area of the outer membrane as it folds internally [28,29]. We used SIM to further characterize the IM structure in mito-bulbs. We stained cells with antibodies directed against either of two IM proteins, ATPase B (ATPB) or Prohibitin 2 (PHB2). Complex V, ATP synthase (stained by ATPB), is thought to be under-represented along the IBM and to be enriched in cristae under normal growth conditions, where it contributes to membrane curvature [30]. PHB2, in complex with Prohibitin 1, is an abundant scaffold component of the IM [31]. The bloated mitochondria of A549 cells show prominent staining of these two proteins in a pattern that is largely concentric with the outer membrane (Fig 2A and 2B). In contrast, PHB2 staining in normal tubular mitochondria (S2A Fig) falls inside Tom20 but appears largely coincident with the mitochondrial matrix with SIM imaging. Both TEM images (Fig 1E) and SIM images (Fig 2A and 2B) of mito-bulbs show that cristae are notably absent from distinct regions in these bloated organelles. Cells in numerous cancers and disease models have been reported to harbor mitochondria with large electron-lucent central regions lacking internal cristae structure [9,32,33]. While nucleoid staining has not been done routinely in these studies, it is possible that these matrix regions may commonly contain clusters of mtDNA nucleoids as shown here.

### Mito-bulbs appear stable for hours

**Fig 3:**
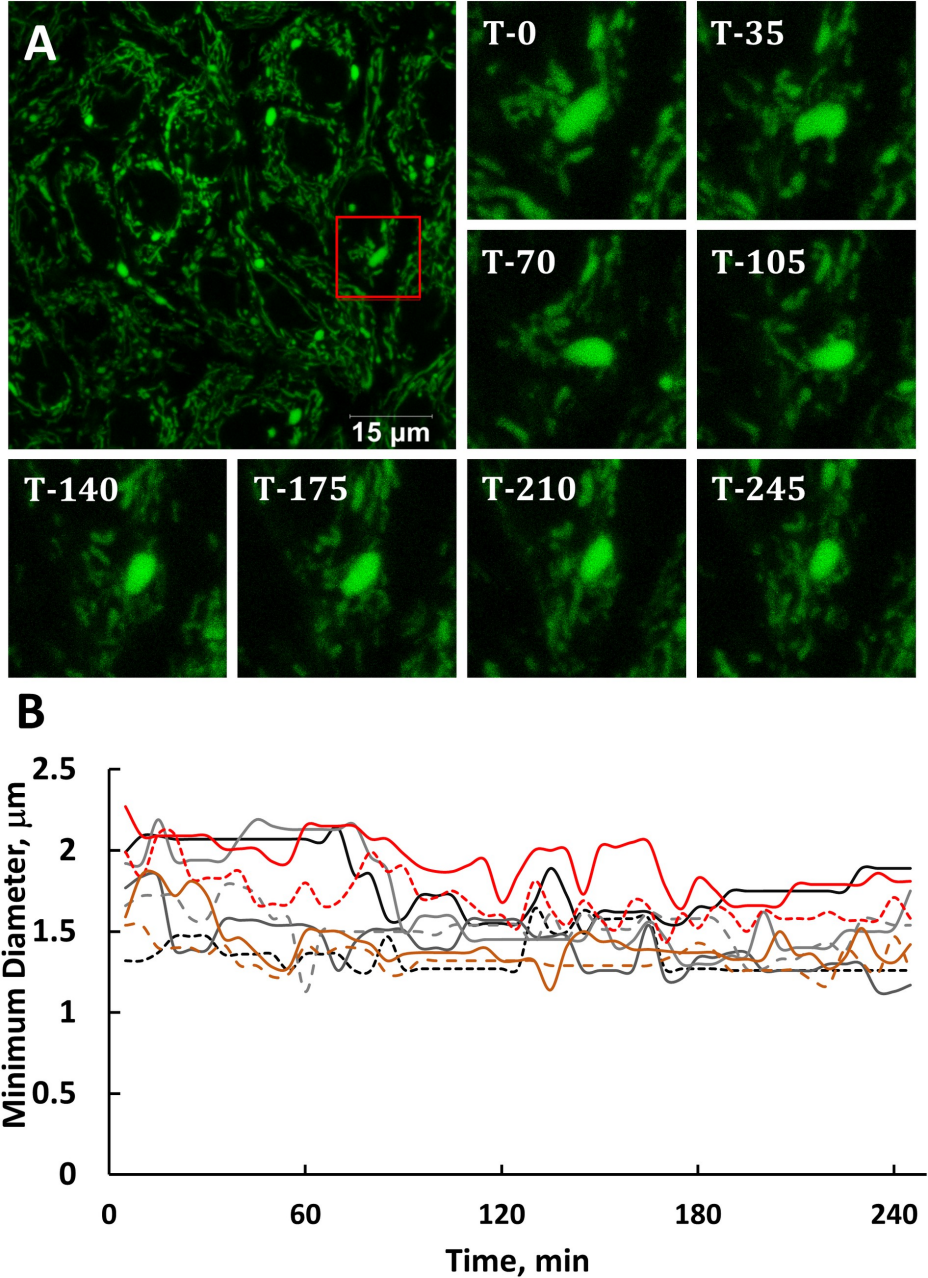
Individual mito-bulb organelles persist over the course of more than 4 h. **A**. Single image frame shows RPMI-adapted A549 cells expressing MLS-EGFP imaged for over 4 h at 5 min intervals via live, z-stack, confocal microscopy. The ROI (red box) denotes a swollen mitochondria, displayed throughout image collection at the indicated 35-min intervals. Scale bar is 15 μm. Leica SP8, 63x, 0.75x zoom, 5% CO_2_, 37⁰C, 95% relative humidity. **B**. Mitochondrial diameters of several individual mito-bulbs are plotted with respect to time, measured at 5 min intervals, showing stability over the course of more than 4 h. Graph represents the measure of the smaller elliptical diameter with respect to time for each mito-bulb measured in A.

Mitochondrial swelling has been reported in numerous settings [34,35] but rarely have swollen mitochondria been examined in a dynamic context especially when known to contain large amounts of mtDNA [14,36]. To gain a better understanding of the dynamics and stability of mito-bulb like mitochondria in A549 cells we generated cells that stably express a mitochondrially-targeted EGFP (MLS-EGFP) to monitor these organelles in a live cell microscope chamber. Confocal z-section stacks of 10 slices, spanning a depth of 2.25 μm, were captured every 5 min for a duration of more than 4 h. Fig 3A shows a representative section and time frames at 35 min intervals from one such video series. Measures of mitochondrial diameter across the smaller of the two axes of bloated elliptical organelles are plotted for 9 individual mito-bulbs in Fig 3B. S1 and S2 Videos show maximum projection time-lapse recordings of an entire field and of a magnified sub-region. The apparently swollen mitochondria have some mobility and flexibility of shape but persist throughout the imaging process and appear to be relatively stable in both position and size over more than 4 h. We did not observe events where elongated mitochondria collapsed into spheroids. Although we limited these recordings to 4 h for technical reasons, it is likely that the bloated mitochondria persist for longer intervals.

### Most bloated mitochondria maintain a membrane potential but feature a more oxidative environment

The electron transport chain of healthy mitochondria maintains a membrane potential of approximately 150 mV across the inner membrane [37]. Since we observed that the inner membrane of swollen mitochondria lacks extensive cristae development, we sought to determine whether the apparently swollen mitochondria maintained membrane potential comparable to that of normal, tubular mitochondria in the same cell. We loaded cells expressing MLS-EGFP with the potentiometric dye tetramethylrhodamine methyl ester (TMRM) to monitor the mitochondrial dye concentration under conditions permitting the green fluorescent protein signal to be used to normalize the signal generated by mitochondria of widely different sizes.

**Fig 4:**
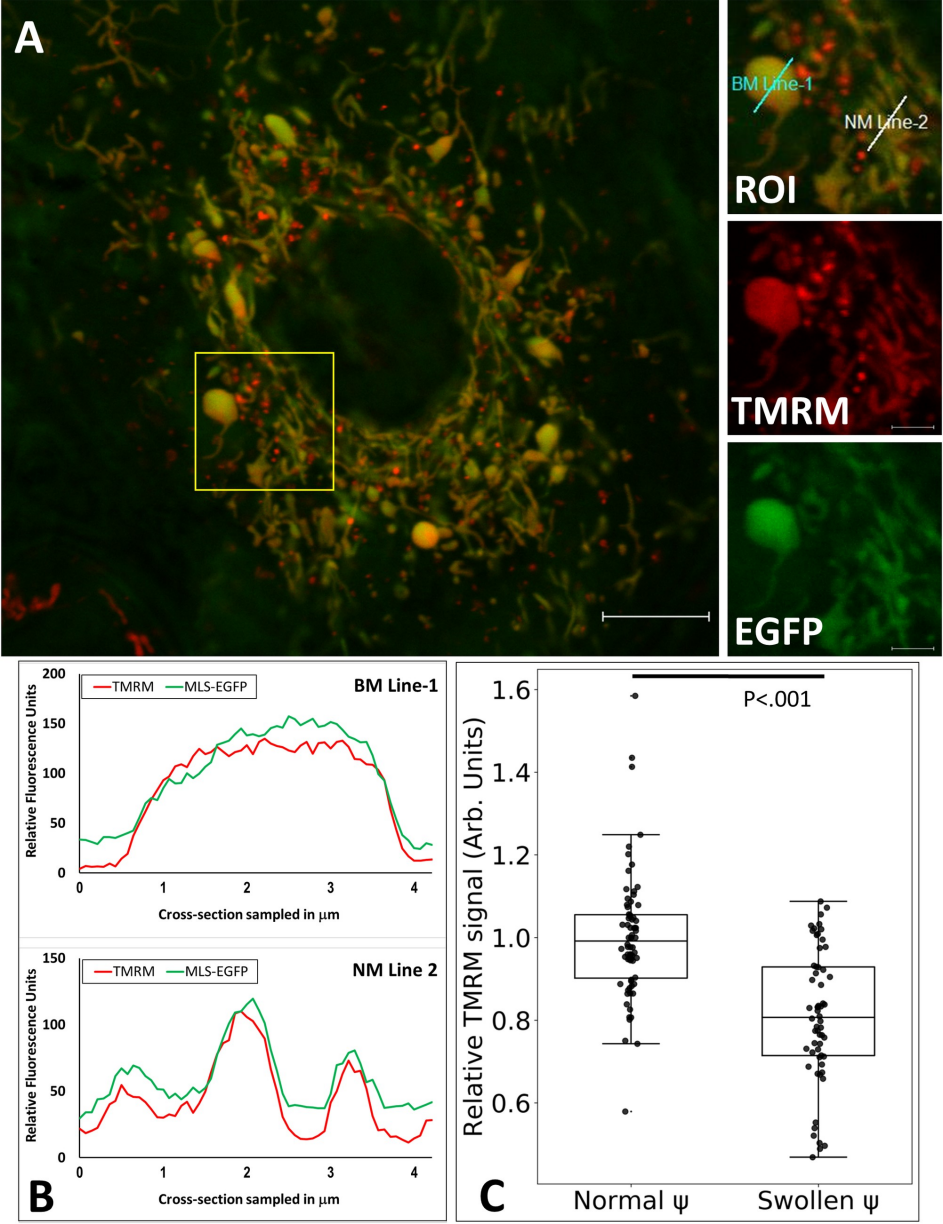
Assessment of the relative membrane potentials of bloated and normal morphology mitochondria via live confocal microscopy. **A**. RPMI-adapted A549 cells expressing MLS-EGFP were loaded with TMRM as described in Materials and Methods. Cells featuring mito-bulbs were imaged and analyzed by tracing the red and green channel signals through regions featuring either mito-bulb or normal tubular morphology mitochondria. Panels on the right show the enlarged ROI boxed in A to indicate the locations of sampling lines and the resolved colors. Scale bar is 10 μm. Leica SP8, 63x, 5% CO_2_, 37⁰C, 95% relative humidity, operating in photon-counting mode. **B**. Sample trace data from the TMRM and EGFP channels. The upper panel depicts a trace through a mito-bulb, “BM Line-1” in A. The lower panel depicts a trace bisecting through three mitochondria with normal morphology, “NM Line-2” in A. Traces follow lines from the lower left to upper right. **C**. The relative TMRM staining intensity normalized to matrix GFP is shown for normal tubular and swollen, mito-bulb mitochondria. Confocal microscopy images from 24 cells, over 3 separate experiments, totaling 132 independent 5 μm mitochondrial traces were processed from data similar to that displayed in A and B. The boxplot shows median and quantile values. Welch’s t-test, t = 6.96, p < .0001.

Due to the differences in membrane architecture and surface to volume ratios between normal morphology and swollen mitochondria we did not attempt to measure the absolute transmembrane voltage potential, only the relative values for swollen and tubular mitochondria (Fig 4). Because expressed MLS-EGFP is used to normalize the TMRM signal, relative comparisons are only valid within the same cells. TMRM-based assessment of 24 cells, imaged over 3 independent experiments, produced trace data through 132 independent mitochondria. Quantitative cross-sectional intensity traces through both mito-bulbs and control neighboring tubular mitochondria were generated, normalized, and analyzed. (Fig 4B). Our data show that the average TMRM:EGFP ratio of the swollen mitochondria compared to that of tubular mitochondria, within the same cell, is reduced by approximately 20% (p < .0001) (Fig 4C). Overall, most A549 cell mito-bulbs maintain a considerable, but decreased membrane potential evidently influenced by the seemingly suboptimal inner membrane arrangement. The apparently altered membrane potential can be explored in more detail using ionophores and respiratory complex inhibitors, but we consider this beyond the scope of the current manuscript. We noted some variability in the relative intensity of TMRM fluorescence in cross-sections of swollen mitochondria. Structural and respiratory inner membrane heterogeneity of normal mitochondria has also been documented recently in tubular mitochondria [38]. During these experiments, we noted a few rare occurrences of apparently depolarized mito-bulbs, which were not included in this statistical analysis. These may reflect serious mitochondrial dysfunction or instances of transitory membrane potential fluctuation [38]. These depolarized organelles were not sufficiently common to permit further study at this time.

**Fig 5:**
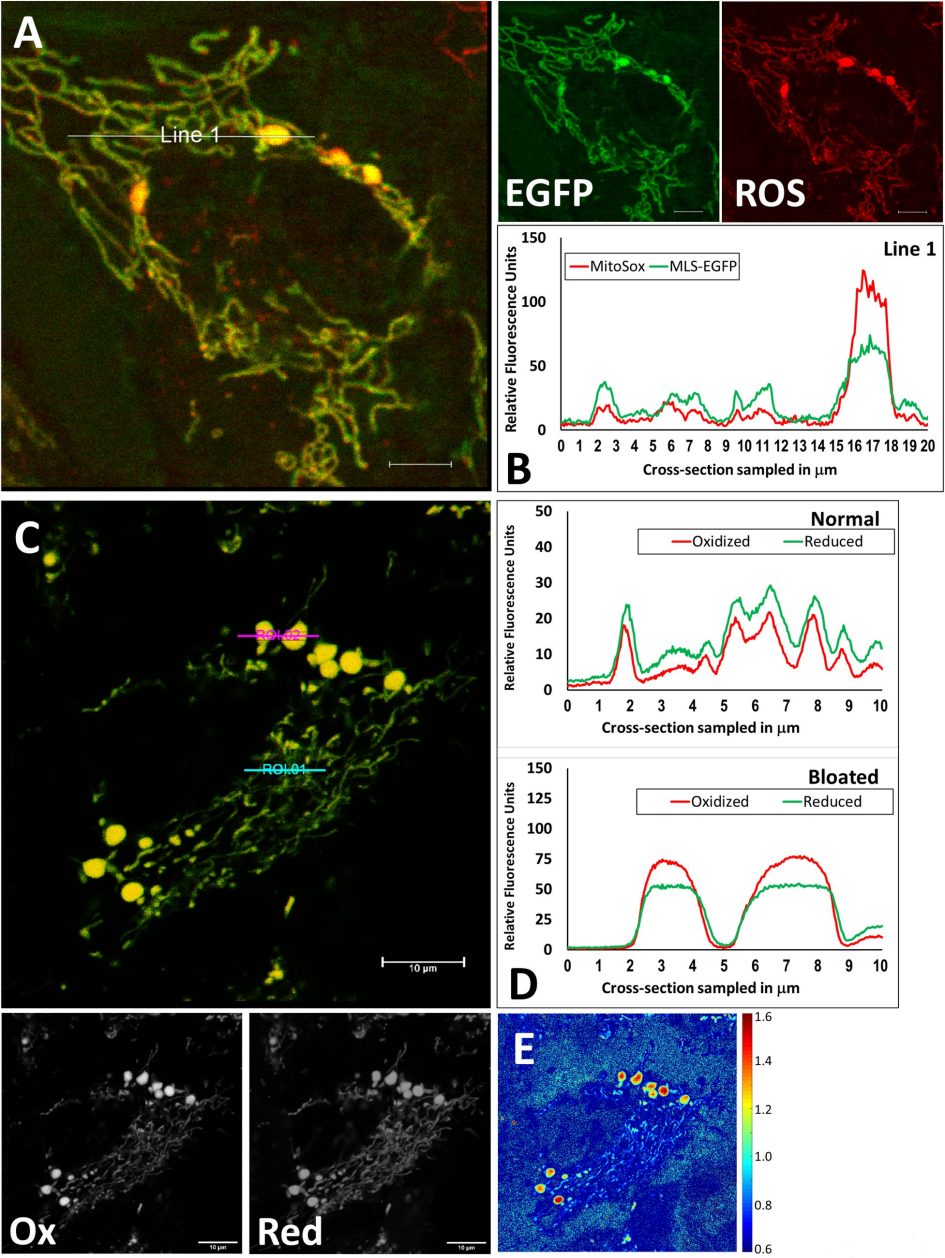
Swollen mitochondria exhibit a more oxidative enviroment than mitochondria with normal morphology. **A.** Representative confocal image of the ROS signal generated by MitoSOX (red) accumulating in A549 cells expressing MLS-EGFP (green). Scale bars are 5 μm. Leica SP8, 63x, 5% CO_2_, 37⁰C, 95% relative humidity, operating in photon-counting mode. **B.** Trace derived from “Line-1” in A that intersects both normal and bloated mitochondria. Line is 20 μm and traces extend from left to right. **C.** Confocal image representing the oxidation state of mito-roGFP in A549 cells. The 525 nm emission intensity signals induced by 405 nm or 488 nm excitation, represent oxidized and reduced protein, respectively, are superimposed in the main panel and resolved individually in panels directly below. Scale bars are 10 μm. Leica SP8, 63x, 5% CO_2_, 37⁰C, 95% relative humidity, operating in photon-counting mode. **D.** Linear traces show the 525 nm fluorescence intensities for the oxidized (red) and reduced (green) forms of mito-roGFP2 in ROIs 1 and 2 of panel C bisecting normal and bloated mitochondria, respectively. **E.** Two-dimensional heatmap of the oxidized:reduced ratio of mito-roGFP.

Mitochondrial metabolism invariably generates reactive oxygen species (ROS) [39], largely due to spillage of electrons from the electron transport chain [40]. We compared ROS production rates in swollen mitochondria with those of normal tubular organelles, initially using MitoSOX, a commonly used ROS indicator that reacts with singlet oxygen, superoxide and peroxynitrite to generate red fluorescence. This showed that prominent swollen mitochondria appeared to accumulate considerably more oxidized MitoSOX (normalized to MLS-EGFP) than the tubular mitochondria (Fig 5A and 5B). However, we did not rely exclusively on this method due to concern that the oxidized MitoSOX is an ethidium derivative that fluoresces upon DNA binding so that the much higher content of mtDNA in swollen mitochondria might generate an artifactually-high fluorescent signal.

To provide an independent measure of mitochondrial ROS, free of such artifacts, we employed a ratiometric, thiol-based ROS-sensitive protein targeted to the mitochondrial matrix, mito-roGFP2 [41,42], to quantitatively assess the redox state of the bloated mitochondria relative to normal mitochondria in the same cell. Fluorescence emission at 525 nm of the oxidized and reduced forms of this protein can be selectively excited by 405 or 488 nm light, respectively. We inserted this reporter in a doxycycline-inducible lentivirus vector expressed in RPMI-adapted A549 cells. Fig 5C shows a representative example of the images obtained from 18 cells, derived over three independent experiments. It is apparent that the signal for the oxidized reporter in the 405 nm-excitation channel (red) is noticeably higher than that of the reduced protein (488 nm excitation; green) for swollen mitochondria as compared to most of the normal tubular mitochondria within recorded cells. This is demonstrated by tracings through two ROIs (Fig 5D), which show a higher ratio of oxidized-to-reduced protein in mito-bulbs relative to tubular mitochondria in the same cell. To visualize the results more broadly, we used a MATLAB script to display the ratio of the oxidized:reduced image intensity as heat-map(s) (Fig 5E). Several additional heat-maps are presented in S3 Fig. Mito-bulbs in these images were reproducibly found to contain a higher ratio of oxidized roGFP2 than the majority of the normal tubular mitochondria. These results more rigorously confirm the preliminary results of the MitoSOX imaging experiment, that prominent mito-bulbs feature a more highly oxidized enviroment than do most tubular mitochondria.

### Nucleoids in clusters are independently active in replication and transcription

**Fig 6:**
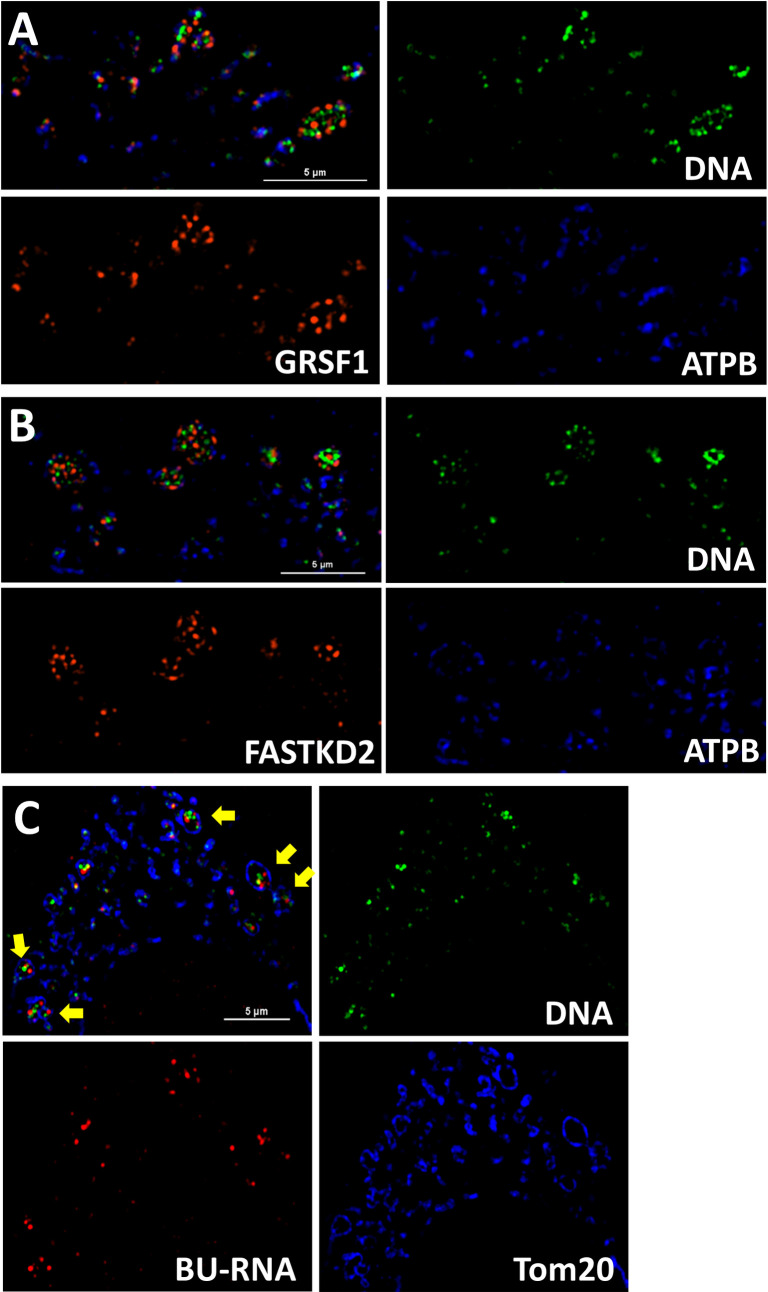
Mito-bulb nucleoid clusters retain transcriptional capability and discrete RNA processing centers. **A.** The MRG marker GRSF1 (red) is observed in individual punctate structures adjacent to mtDNA nucleoids (green) within mito-bulb like structures. Inner membrane marker ATPB is depicted in blue. Scale bar is 5 μm. Image is representative of six specimens. **B.** The MRG marker FASTKD2 (red) resides in individual punctate structures adjacent to mtDNA nucleoids (green) within mito-bulb like structures. Inner membrane marker ATPB is depicted in blue. Scale bar is 5 μm. Image is representative of 25 images collected for seven independent specimens. **C.** The nascent BU-labeled mtRNA (red) is observed in individual punctate structures adjacent to mtDNA nucleoids (green) inside both normal and bloated mitochondrial structures. Outer membrane marker Tom20 is depicted in blue. Arrows indicate swollen mitochondria with multiple foci of nascent mtRNA. Scale bar is 5 μm. Image is representative of 10 images of three independent experiments.

A major goal of our investigations into the unusual morphology of mito-bulbs in A549 cells was to determine the consequences of the clustering of individual nucleoids in a common matrix space. Since individual nucleoids do not fuse into a single large structure within mito-bulbs, they may continue to persist as independent centers for replication and transcription, but their gene products may be expected to cooperate in local mitochondrial biogenesis. In tubular mitochondria, nascent mitochondrial transcripts are processed to generate rRNAs, tRNAs and mRNAs in the immediate vicinity of mtDNA nucleoids within MRG [6] or MIOREX complexes in yeast [43]. Numerous proteins involved in mtRNA processing are thought to localize to MRG [44], including FASTKD2 and GRSF1 as two of the better characterized components. We reasoned that one consequence of nucleoid clustering in A549 cells might be that the transcripts of individual nucleoids could be pooled to facilitate sharing of genetic information between clustered nucleoids. To test this we used SIM to image A549 cells stained with anti-DNA antibodies and either of the MRG markers GRSF1 or FASTKD2. Fig 6A and 6B reveal multiple discrete MRG clustered alongside nucleoids within mito-bulbs and are representative of 28 cells imaged in 8 independent specimens.

To explore the transcription activity in mito-bulbs, we labeled A549 cells with bromouridine for 45 min and imaged nascent mtRNA along with anti-DNA staining for nucleoids and antibodies against the outer membrane protein Tom20 to identify mitochondria. In these experiments labeling must be confined to short intervals to limit the export of nascent BU-labeled RNA from the nucleus. We observed multiple separate BU-labeled RNA granules clustered with discrete nucleoids inside mito-bulbs (Fig 6C). Together these images suggest the hypothesis that nucleoid-MRG pairs function largely as distinct units likely with minimal exchange of RNA transcripts even when multiple nucleoids share a contiguous matrix space, although we cannot rule out the possibility that some exchange of RNA occurs between MRGs.

**Fig 7:**
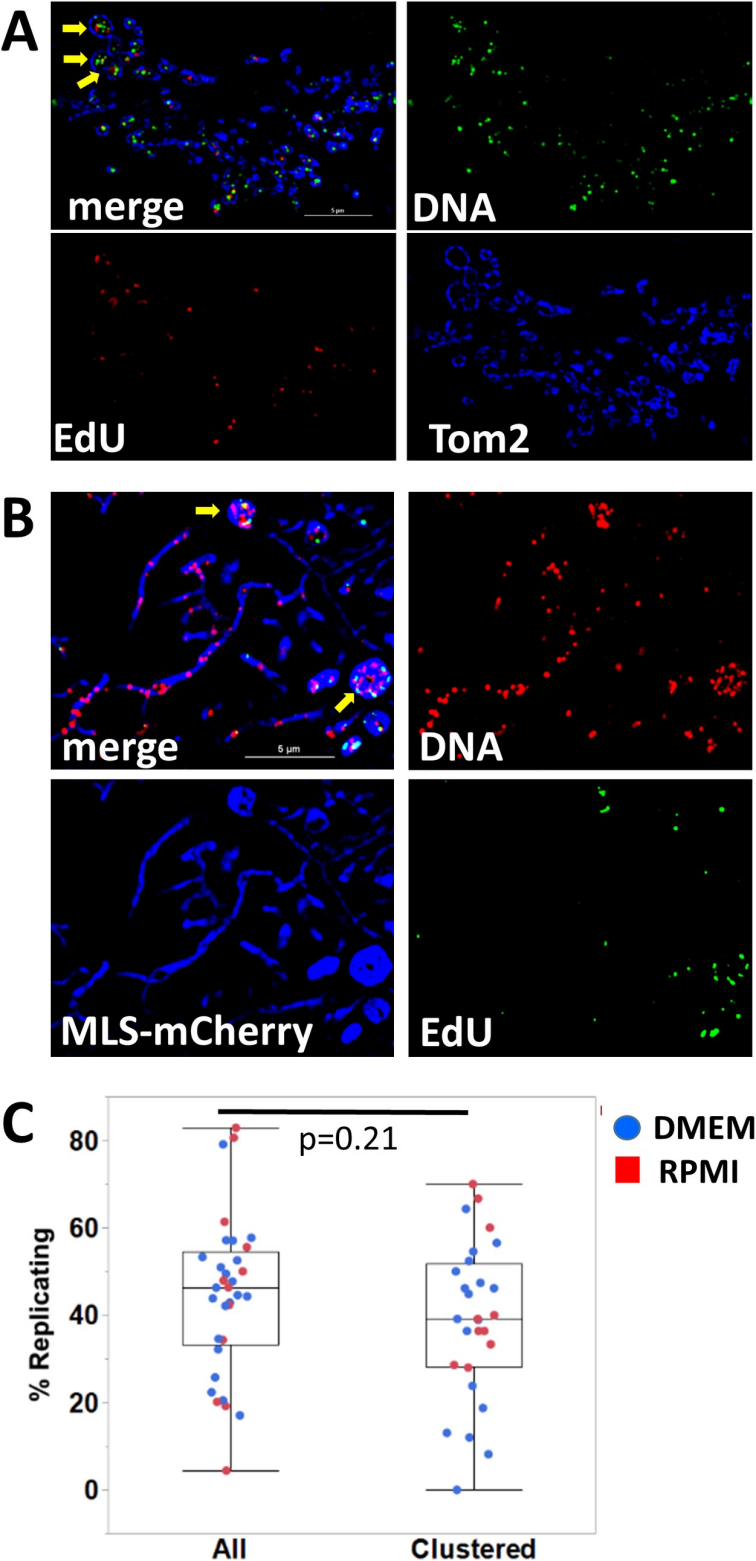
Nucleoids in mito-bulbs are active in mtDNA replication. **A.** DMEM-adapted A549 cells show positive labeling for EdU foci (red) inside mitochondria at mtDNA nucleoids. Replication is active inside mito-bulb like mitochondria indicated by yellow arrows. Cells were stained with antibodies against DNA (green) and Tom20 (blue). Resolved channels are displayed as separate panels as indicated. Scale bar in A is 5 μm. **B.** RPMI-adapted A549 cells expressing MLS-mCherry (blue) were labeled with EdU detected using click chemistry (green) and stained with anti-DNA antibodies (red). Arrows indicate EdU incorporation at nucleoids in bloated mitochondria. Scale bar is 5 μm. **C.** Box-and-whisker plot of the percentage of nucleoids that incorporated EdU in the total population of nucleoids and in the subset of nucleoids clustered in bloated mitochondria. Data for 33 specimens in 4 independent experiments are compiled in Table 2. Boxes indicate means and quantile values for the distributions, which were not significantly different (p = 0.21).

We also investigated the dynamics of mtDNA replication in mito-bulbs. MtDNA molecules in mouse L cells are known to replicate independently and to take an hour to complete replication [45,46], but the factors that limit the frequency of mtDNA replication are poorly understood. We considered that clustered mtDNA molecules in bloated A549 cell mitochondria might have access to a larger pool of replication factors and that an increased rate of replication might contribute to the accumulation of numerous nucleoids in these organelles. Thus, we analyzed mtDNA synthesis by pulse labeling DMEM-adapted cells for 1 h with EdU and detection by conjugation of EdU to a fluorescent dye using click chemistry. These cells were subsequently stained with antibodies against DNA and Tom20 and visualized using SIM, as shown in Fig 7A. In addition, in light of the morphological differences observed when A549 cells are grown in RPMI as opposed to DMEM, we decided to compare the EdU incorporation rates within tubular mitochondria to those of nucleoids in mito-bulbs in cells cultured in RPMI. Therefore, we repeated the EdU labeling experiment in RPMI-adapted cells as they more commonly feature typical tubular mitochondrial morphology alongside occasional mito-bulbs. In this set of experiments we used cells expressing mitochondrial-targeted mCherry to mark the matrix space and continued the pulse labeling for 2.5 h, with a representative image in Fig 7B. The fraction of nucleoids labeled with EdU under either condition is depicted in Fig 7C, where each point represents analysis of an independent image. A considerable variability was observed in the percentage of nucleoids labeled with EdU, with no significant difference in the variance of the datasets for total or clustered nucleoids. In both media conditions, we observed that some nucleoids in clusters incorporated label while others did not, indicating that nucleoids in clusters replicate independently. Table 2 shows the numbers of total and EdU-positive nucleoids in 33 images from 4 experiments, with separate totals for clustered nucleoids in bloated mitochondria. Statistical analysis of the results for nearly 4000 nucleoids showed that the fraction engaged in replication was not statistically different for the 731 clustered nucleoids (36.8+3.3%) as compared to the total population (40.5+3.1%). Thus, we conclude that clustered nucleoids in bloated mitochondria in A549 cells are not generally more likely to engage in replication.

**Table 2:** Nucleoids in tubular and swollen mitochondria replicate with similar frequency.

	All nucleoids		Clustered	
	Nucleoids	EdU+	Nucleoids	EdU+
DMEM	2614	1030	483	161
RPMI	1270	543	248	108
Total	3884	1573	731	269
Mean		40.5%		36.8%
SE		3.1%		3.3%

P = 0.21, 95% confidence level.

A549 cells grown in DMEM or RPMI were labeled with 50 μm EdU for 1 h or 2.5h, respectively in 4 independent experiments as described in Materials and Methods and the legend to Fig 7. 33 images from different cells were analyzed with Nikon Analyst software to count total nucleoids and EdU foci. Mitochondria with a minimal diameter of 1.2 μm containing at least three nucleoids were scored as swollen organelles and the total nucleoids and EdU foci were counted using Analyst software with manual supervision. ANOVA was used to calculate the weighted mean and standard error (SE) for the total population and for nucleoids in clusters. Student’s t-test showed that the fraction of nucleoids replicating in swollen mitochondria containing clusters of >3 nucleoids was not statistically different from the overall population (p = 0.21, 95% confidence limit).

### Incidence of mito-bulbs correlates with elevated glucose and/or glutamine media concentration

**Fig 8:**
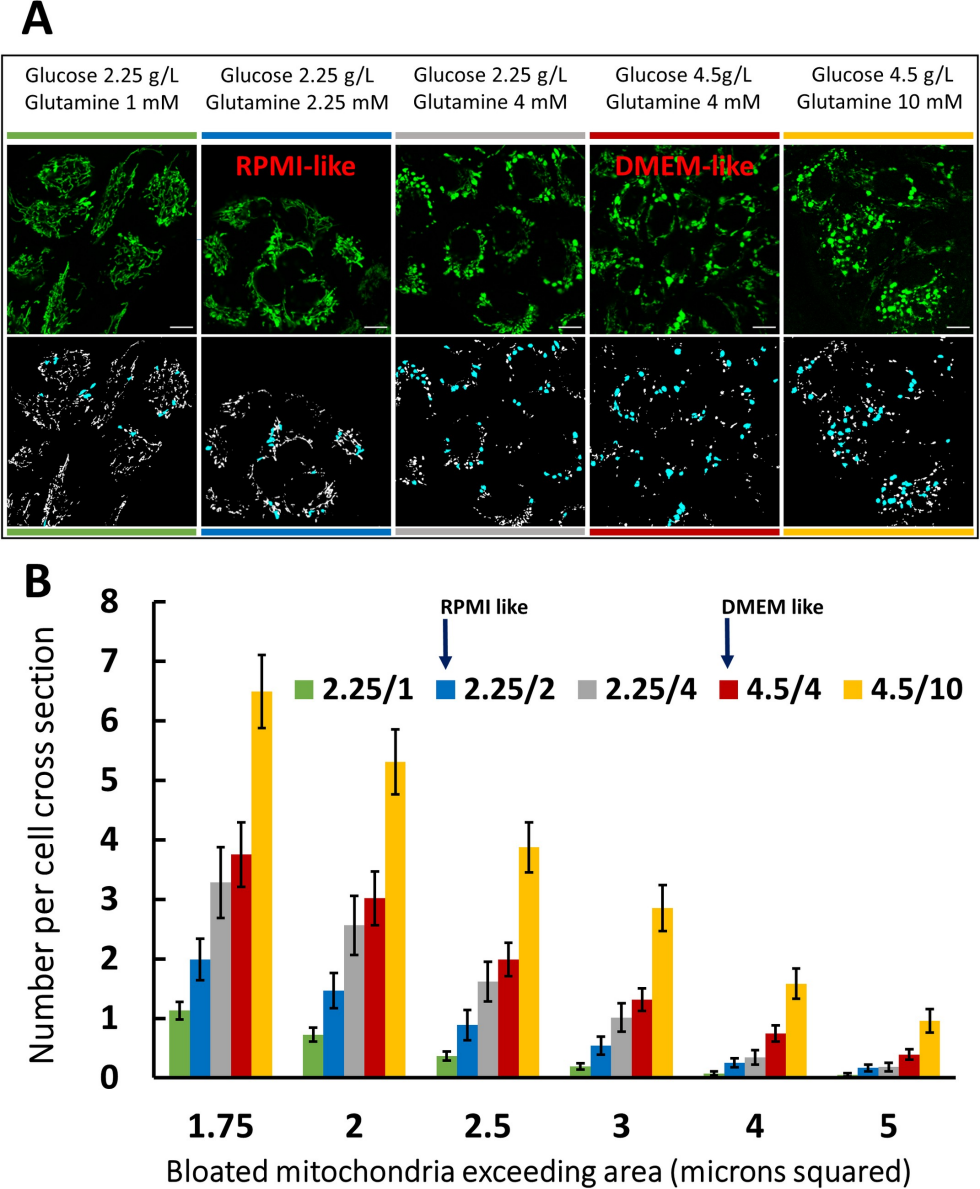
Incidence of mito-bulbs correlates with elevated glucose and/or glutamine concentration. **A.** Five columns show representative images of MLS-EGFP A549 cells grown in basal DMEM media with varied glucose and/or glutamine content as noted. Below each image is the binarized mask of each image highlighting mito-bulbs with cross-sectional areas above 2 μm^2^ in blue. Scale bars are 10 μm in each image. Leica SP8, 63x, 2.5x zoom. Color-coded bars above and below the images are keyed to the histogram in B. **B.** Histograms showing the average number of mito-bulbs per cell in each condition listed. The bar graph shows the mean number (+/- standard deviation) of mito-bulbs per cell exceeding the indicated threshold in μm^2^ under each of five media conditions. A total of 95 images (total of 958 cells) were analyzed to generate this graph. The number of images taken and analyzed was roughly equivalent among all the conditions, with the exception of a slightly higher number taken and analyzed for the lowest glucose/glutamine condition to compensate for the lower incidence of mito-bulbs.

Swollen mitochondria with clustered nucleoids are observed in A549 cells cultured in both RPMI and DMEM, but at a higher frequency in the latter (S1 Fig). This difference in population incidence is important as we have shown that mito-bulbs likely maintain a lower membrane potential, feature a more oxidized internal enviroment and have a higher mtDNA content and replication frequency. These two media differ in several ingredients, including the concentrations of glucose and glutamine. DMEM has roughly twice the concentration of each of these nutrients as compared to RPMI (4500 vs 2000 mg/l glucose; 4 vs 2 mM glutamine, respectively). To quantify the incidence of mitochondrial swelling we collected series of confocal images of MLS-EGFP A549 cells grown in basal glucose and glutamine free DMEM medium supplemented with varied concentrations of glucose and glutamine. We employed a watershed algorithm to quantify mito-bulbs as described in Materials and Methods. As in S1 Fig, we set variable cutoffs for “minimum cross-sectional area”, showing that at any given size threshold, the frequency of occurrence of swollen mitochondria increases with increasing concentrations of glutamine and possibly glucose (Fig 8).

### Mito-bulb incidence can be influenced by TGFβ pathway agonists and antagonists

**Fig 9:**
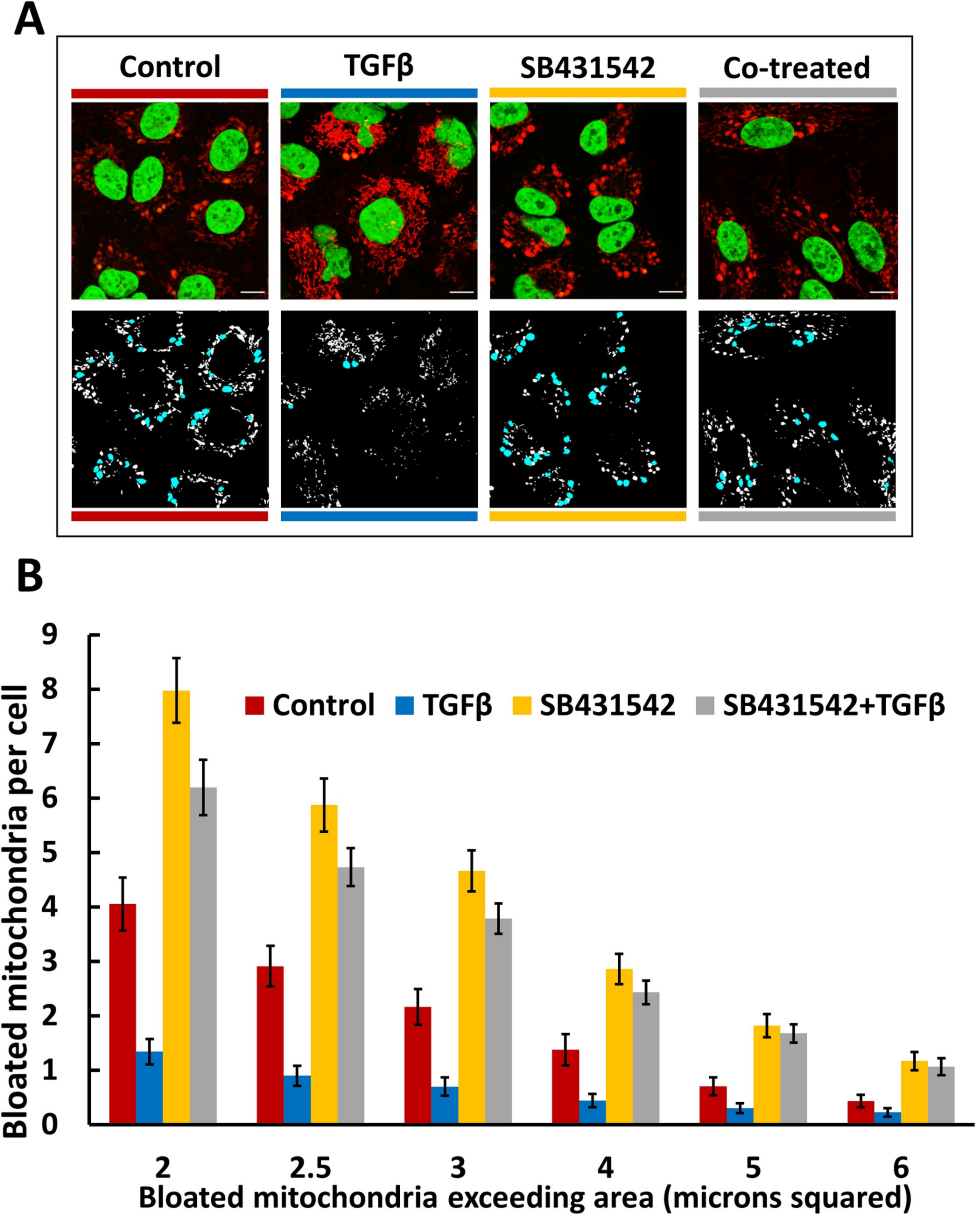
Mito-bulb incidence can be influenced markedly by inhibition of the TGFβ pathway. **A.** Four columns feature representative images of MLS-EGFP, DMEM adapted A549 cells, for each treatment condition assessed. Cells were treated for 4.5 days with either vehicle control, 5 ng/ml TGFβ, 10μM SB431542, or with both compounds simultaneously, from left to right, respectively. Mitochondrial networks, as visualized via MLS-EGFP are depicted in red and Hoechst stain is depicted in green. Scale-bars are 10 μm. Leica SP8, 63x, 2.5x zoom. **B.** Graph of the average number of mito-bulbs per cell (+/- standard deviation) exceeding the indicated threshold for each condition specified, as in Fig 8. A total of 72 images (total of 495 cells) were analyzed to generate this graph. The number of images taken and analyzed was roughly equivalent among all the conditions.

The A549 cell line is an accepted model for the study of EMT induced by TGFβ treatment [21,47]. Our preliminary experiments showed that passaging A549 cells adapted to either RPMI or DMEM in the presence of 5 ng/ml TGFβ results in drastic morphological changes, including at the mitochondrial network level, within 4–10 days. After 5 to 10 days almost all the cells generally appear large, flat and upregulate markers characteristic of EMT [20]. The mitochondrial network in these TGFβ-treated cells is comprised of many interconnected tubular mitochondria with a striking reduction in the incidence of mito-bulbs. We also tested whether addition of TGFβ pathway antagonist SB431542 [48] would influence the incidence of mito-bulb mitochondrial morphology. We treated DMEM-adapted A549 cells with either vehicle control, 5 ng/ml TGFβ, 10 μM SB431542, or both reagents simultaneously, for 4.5 days to analyze the intermediate-term effects of these agents on mitochondrial morphology. Fig 9 reveals that treatment with TGFβ significantly decreased the incidence of mito-bulbs while exposure to 10 μM SB431542 promoted formation of mito-bulbs and substantially blocked the effects of TGFβ on mitochondrial morphology. This was not due to toxicity of SB431542 as culture and passaging of A549 cells for 4 weeks with 10 μM SB431542 did not reveal any overt toxicity (not shown). Previous studies have reported that SB431542 increases mitochondrial size in isolated organelles, and that SB431542 can act as a potent anti-tumor agent only in TGFβ-sensitive cell lines [49,50]. However, to our knowledge the influence of SB431542 on mito-bulb formation has not been investigated.

## Discussion

### Intracellular mitochondrial heterogeneity in A549 cells

The filamentous nature of mitochondria is inherent in their name as suggested by Benda in 1898 (“mitos”, thread; as cited [51]). Nevertheless, there is extensive heterogeneity in mitochondrial form and function, particularly between different cell types. It is more unusual to find examples of considerable heterogeneity of mitochondria *within* single cells, as in the example we describe of A549 cells. We show that these cells can simultaneously host elongated tubular mitochondria alongside swollen spheroidal organelles with a far larger minimal diameter. Due to this heterogeneity and to the difficulty of biochemically separating these two classes of mitochondria, we have confined our present study largely to microscopic characterization of the apparently swollen mitochondria which we have termed “mito-bulbs” following [14]. These mito-bulbs might also be referred to by an alternate organelle name also attributed to Benda [51], “chondriosphären” or “sphere-like granules”.

We show here that the mito-bulbs in A549 cells can attain diameters of a few microns, but contain greatly expanded internal matrix space lacking the usual extensive cristae in-foldings. This is documented in Fig 2 both with the inner membrane marker Prohibitin 2, which is well represented in the IBM and cristae, as well as with ATP synthase B, which is expected to occur at higher levels at the curved edges of cristae membranes owing to the membrane-bending tendency of ATPase dimers [52]. We show that these mito-bulbs have a slightly lower membrane potential and have a more oxidative environment than tubular mitochondria in the same cell (Figs 4 and 5). This reduced membrane potential is nevertheless sufficient to permit the potential-dependent import of proteins, including our MLS-EGFP and mito-roGFP2 reporters. We were concerned that the “mito-bulbs” might be transitory structures resulting from fluctuations in mitochondrial shape as Tauber et al. (2013) have suggested [11], but we found that individual swollen mitochondria persist for hours (Fig 3).

Current models for mitochondrial turnover suggest that dysfunctional segments of tubular mitochondria can be eliminated by fission events producing smaller organelles that are incapable of re-joining the mitochondrial reticulum through fusion [8], but are susceptible to degradation by mitophagy [53]. There are two reasons why the A549 cell mito-bulbs may not be amenable to culling by this approach. First, it is likely that the enlarged mitochondria are not susceptible to fission by the normal mechanism in which Drp1 (DNML1) is recruited to the mitochondria to form a division ring because this is adapted for cleavage of a narrower tubule [54]. Second, the intact mito-bulbs may also be too large to be enclosed in an autophagic vacuole. To date we have not seen evidence of fission of the enlarged mitochondria to increase their susceptibility to mitophagy. A549 cells have been shown to have low levels of Drp1 bearing a greater proportion of inactivating S637 phosphorylated residues than of activating S616 phosphates [55], which does not favor mitochondrial fission.

The incidence of mito-bulbs can be altered substantially by changes in culture conditions. First, we noted a higher incidence of this morphology in cells grown in DMEM as opposed to RPMI medium, both in an environment with 20% oxygen, which rules out hypoxia as a contributing factor [56]. This morphology difference was reversible over a period of a few days upon switching to the alternate medium and mito-bulb incidence was enhanced in medium supplemented to contain higher concentrations of glucose and/or glutamine (Fig 8). However, there are other differences in the formulations of DMEM and RPMI that may also contribute to the differences we observed in mitochondrial morphology, such as the increased concentration of calcium and lower concentration of phosphate in DMEM. We explored the impact of changes in glucose and glutamine concentrations because of the known reliance of cancer cells on oxidative glucose metabolism, the Warburg effect, and on glutamine to supply anaplerotic substrates for the TCA cycle and the pentose phosphate pathway [57,58]. While additional factors may be explored in future research, our results suggest that nutritional excess may contribute to the formation of mito-bulbs. In line with this, induction of stress by serum deprivation or amino acid starvation [59,60] is known to lead to mitochondrial hyperfusion.

We found that a more dramatic transition in mito-bulb incidence can be achieved by manipulating the TGFβ pathway. It has been known for some time that A549 cells differentiate towards a mesenchymal phenotype in response to TGFβ [61]. We show that this correlates with a decrease in mito-bulb incidence and an increase in normal tubular morphology. Conversely, treatment with the TGFβ pathway inhibitor SB431542 induces a remarkable increase in mito-bulb formation (Fig 9). It may be that the potential anti-cancer activity of SB431542 is enhanced by its dramatic effects on mitochondrial morphology, with the attendant decreased membrane potential and increased ROS production. A complete analysis of the status of proteins involved in mitochondrial fusion and fission and of the overlying interactions with cytoskeletal elements [62] and ER tubules [63] that help govern mitochondrial dynamics in this setting is beyond the scope of our current investigations.

### A549 mito-bulbs provide a remarkable example of mtDNA nucleoid clustering

Our inquiry into mitochondrial morphology in A549 cells is focused on the observation that the large mito-bulb structures clearly hosted multiple independent mtDNA nucleoids sharing a single enlarged matrix space. This provides a stark contrast to the more typical setting where single nucleoids are evenly spaced in tubular mitochondria. Within such tubular mitochondria, the relative isolation of individual nucleoids makes it likely that the gene products of a single mtDNA molecule are more likely to be located in its local vicinity [4], providing a linkage between the mitochondrial genotype and the local phenotype. This linkage is abrogated when nucleoids cluster dramatically in mito-bulbs since multiple genomes contribute to the local environment.

Our imaging studies clearly show that the multiple mtDNA nucleoids maintain their independent structure in the common environment of mito-bulbs. This is a significant observation since some models of nucleoid structure have suggested that the major mtDNA binding protein, TFAM, might be capable of fusing multiple mtDNA molecules into larger nucleoid structures. First, Kukat et al. [64] suggested that TFAM might employ its two HMG-box DNA binding motifs to link two separate strands of duplex DNA. The observation of distinct nucleoids in swollen mitochondria of A549 cells implies that this is not sufficient to promote coalescence of multiple genomes into single enlarged complexes. Second, TFAM has recently reported to have some ability to form molecular condensates [16]. Such a liquid-liquid phase separation might also be expected to be capable of fusing two mtDNA molecules into larger structures, which we have also not observed. We found that individual nucleoids also appear to maintain functional independence, engaging in replication and transcription as discrete entities.

Symmetrical transcription of mtDNA generates RNA copies of both strands that require endonucleolytic cleavage to produce rRNA, mRNA and tRNA products. This processing is initiated in the vicinity of nucleoids, continues in nearby MRG [6], and involves selective degradation of extensive anti-sense sequences by the degradosomes [65]. In principle, a moderate degree of nucleoid clustering may serve to permit multiple mtDNA genomes to contribute transcripts to shared RNA processing factories. It is possible that the reported ability of the MRG protein FASTKD2 to engage in formation of molecular condensates capable of fusing [66] might contribute to the formation of large centralized RNA processing centers in mito-bulbs. This may be a beneficial response to organellar stress under some circumstances. However, we find that larger mito-bulbs contain multiple independent foci of newly synthesized mtRNA and MRG. This suggests that the size of individual MRG may be constrained by factors that are not yet well-understood. Our results do not rule out the possibility that individual MRG may process transcripts emanating from more than one discrete nucleoid.

We also show in two settings that mtDNA replication is active in mito-bulbs (Fig 7). Short-term (1 or 2.5 h) labeling with EdU revealed no statistically significant difference in replication rates for clustered genomes as for isolated ones. However, the frequency of nucleoid labeling appears higher than expected in these experiments, suggesting that synthesis of 7S DNA in mtDNA D-loops, which is known to account for a large fraction of nascent mtDNA synthesis due to turnover [67], might contribute to the apparent replication frequency. We did observe some striking examples of elevated replication frequency in unusually large mito-bulbs, as in Fig 7B. This is consistent with the hypothesis that increased replication may contribute to the abnormal accumulation of mtDNA observed in these bloated mitochondria. This imbalanced replication may eventually lead to increasing mitochondrial dysfunction contributing to the reduced membrane potential and increased ROS we have documented in mito-bulbs. Whether this progresses to such a degree that oxidative damage or the failure to import proteins leads to a demise of these organelles remains to be seen.

## Materials and methods

### Key reagents

**Table:** 

**Antibody reagents**			
**Antigen**	**Species**	**Vendor**	**Dilution**
DNA	mouse	Chemicon; MAB030; IgG2a	1–12,000
FASTKD2	rabbit	Proteintech; 17464-1-AP	1:1000
GRSF1	rabbit	Sigma; HPA036985	1:1000
ATPase B	mouse	Abcam; ab14730; IgG1	1:1000
Tom20	rabbit	Santa Cruz; sc-11415	1:1000
PHB2	mouse	Proteintech; 66424-1-IG	1:1000
bromouridine	mouse	Sigma; 11170376001; IgG1	1:100
anti-rabbit AF488	goat	Thermo-Fisher; A11008	1:1000
anti-rabbit AF568	goat	Thermo-Fisher; A11036	1:1000
anti-rabbit AF594	goat	Thermo-Fisher; A11032	1:1000
anti-rabbit AF647	goat	Thermo-Fisher; A21244	1:1000
anti-mouse AF488	goat	Thermo-Fisher; A11001	1:1000
anti-mouse AF568	goat	Thermo-Fisher; A11004	1:1000
anti-mouse AF594	goat	Thermo-Fisher; A11037	1:1000
anti-mouse AF647	goat	Thermo-Fisher; A21235	1:1000
anti-mouse IgG2a AF488	goat	Jackson Immuno; 115-545-206	1:1000
anti-mouse IgG2a AF647	goat	Jackson Immuno; 115-605-206	1:1000
anti-mouse IgG1 AF555	goat	Thermo-Fisher; A21127	1:1000
anti-mouse IgG1 AF647	goat	Jackson Immuno; 115-605-205	1:1000
Anti-mouse Fluoronanogold	goat	Nanoprobes; 7202	1:80
**Plasmids**			
pLenti CMV rtTA3G Blast		Addgene #31797	
pLenti CMVTRE3G EGFP Puro		Addgene #27570	
pLANS mCherry		Addgene # 55102	
pLANS roGFP2		Addgene #49437	
pEGFP-N1		Takara	
pLenti CMV EGFP		Addgene #17448	
psPax2		Gift from Dr. Ken-Ichi Takemaru	
pMD2.G		Gift from Dr. Ken-Ichi Takemaru	
**Cell culture media and components**			
DMEM		Thermo-Fisher; 11965–092	
RPMI		Thermo-Fisher; 11875–093	
Phenol free DMEM -glucose -glutamine		Thermo-Fisher; A14430-01	
Phenol free RPMI -glutamine		Thermo-Fisher; 32404–014	
L-Glutamine		Thermo-Fisher; 25030–081	
MitoSox		Thermo-Fisher; M36008	
TMRM		Thermo-Fisher; I34361	
TGFβ		R&D Systems	
SB431542		AbMole Biosciences	
catalase		Sigma; C3155-50MG	
cysteamine		Sigma; 30070-10G	
Glucose oxidase		Sigma; G2133-50KU	
**Kits**			
Lipofectamine 2000 +Plus		Thermo Fisher	
HQ silver enhancement		Nanoprobes; 2012	
Click-iT™ EdU-AF488		Thermo Fisher; C10337	
Click-iT™ EdU-AF647		Thermo Fisher; C10640	

### Cell culture and transfection

A549 cells were initially obtained from Dr. John Haley (Stony Brook, NY, USA) and were verified by ATCC testing (Manassas, VA, USA). Cells were maintained in either DMEM or RPMI, or phenol-red free DMEM modified to contain specified glucose/glutamine concentrations (all from Thermo Fisher). Media contained 10% fetal bovine serum (Atlanta Biologics, R&D Systems) and 1% Penicillin-Streptomycin cocktail (Thermo Fisher) for cell growth in a humidified 5% CO_2_ incubator at 37 ⁰C. Cell replication rates in DMEM and RPMI were monitored by triplicate sampling of parallel cultures at intervals using hemocytometer cell counts. Where indicated, TGF-β1 was used at a concentration of 5 ng/ml. Cells were grown on glass bottomed MatTek dishes (P35GC-1.5-14-C) for imaging applications.

Lentiviral vectors used include pLenti CMV rtTA3G Blast and pLenti CMVTRE3G EGFP Puro with both resembling the Tet-On-3G system (Takara Bio). The expression vector was modified to remove extraneous NotI sites to permit cloning of gene element cassettes between unique AscI and NotI sites and between NotI and SbfI sites to generate pLANS-TRE-MLS-mCherry and pLANS-TRE-MLS-roGFP2. mCherry and roGFP2 were cloned from Addgene plasmids indicated in the table, using appropriate PCR primers.

Lentiviruses were made as previously described in Lenti-X Hek-293T cells (Takara Bio) essentially as described [68]. Transductions of A549 by pLANS-TRE constructs at appropriate viral titer were roughly estimated using parallel control transductions with pLenti CMV EGFP monitored by co-staining using Hoechst 33342 and observation via inverted epifluorescence microscopy. Cell lines requiring doxycycline-inducible expression were co-transduced by desired pLANS-TRE and CMV-rtTA lentiviruses, selected and stabilized using selection with blasticidin and puromycin (Thermo Fisher).

The MLS-EGFP plasmid was derived from pEGFP-N1 (Takara Bio) by inserting the mitochondrial localization signal of DNA POLG2 upstream from and in frame with EGFP to visualize the mitochondrial matrix as described previously [69]. The plasmid was transfected into A549 cells using Lipofectamine 2000 LTX+Plus reagent according to company literature (Thermo Fisher). Cells were selected with G418 (800 μg/ml; Thermo Fisher) and occasionally treated with maintenance doses of G418 (400 μg/ml). Flow sorting was also initially performed to enrich for cells expressing mitochondrially localized EGFP at adequate intensity.

### Immunofluorescence, antibodies and microscopy

Cells were fixed with 4% paraformaldehyde or with 3% paraformaldehyde/0.05% glutaraldehyde (both EM Sciences) and processed for immunofluorescence imaging by SIM, STORM or confocal microscopy as described [70], including treatment with NaBH_4_ when glutaraldehyde was used. Cells were permeabilized with 0.2% Triton X-100 and blocked with 5% normal goat serum in PBS containing 0.05% Triton X-100 for 1 hr. Antibodies used are shown in the table of key reagents. Samples were washed three times for 10 min with PBS containing 0.05% Triton X-100 after each antibody incubation and were routinely post-fixed with 4% paraformaldehyde for longer term storage.

### Confocal laser scanning microscopy

Imaging was performed using a laser scanning Leica TCS SP8 Confocal microscope (Leica Microsystems, Wetzlar, Germany) equipped with white light laser and 405 diode. Images were captured sequentially on fixed samples, and line-by-line for live samples, using the white light laser or 405 diode where appropriate, corresponding to optimized emission spectra of antibodies, fluorescent proteins, and dyes used. For live imaging, the cells were maintained at 37°C and 5% CO_2_ in a humidified microscope incubation chamber (Leica Microsystems, Wetzlar, Germany). Live cell imaging was performed in phenol-free DMEM/RPMI media with glucose/glutamine concentrations adjusted where noted. For channels and applications where signal intensity was not explicitly quantified, hybrid detectors (HyDs) were used to collect signal. For quantitative signal channels/applications images were collected on HyDs operating in photon-counting mode.

### Structural illumination microscopy (SIM)

Structured illumination microscopy (SIM [22]) was performed on a Nikon Ti-E microscope with Coherent 405, 488, 561 and 640 nm lasers, a 100X 1.49NA objective and an Andor iXON3 EMCCD detector controlled by NIS elements AR software with specimens in standard PBS buffer. Laser intensity and exposure time were optimized to occupy 25% of the 14-bit gray scale intensity. Images were reconstructed with illumination modulation contrast of 1.0, high resolution noise suppression 1.0, and out of focus blur suppression of 0.15 or 0.2. 15 fields of 512x512 arrays at 0.06 μm/pixel were acquired for each image. Post-acquisition processing using Nikon AR software was performed to modulate image intensity by adjustment of background cutoff and maximal signal intensity in a strictly linear manner. Morphometric analysis of images was done using thresholding and object identification tools in the NIS Elements AR software.

### STORM microscopy

Stochastic optical reconstruction microscopy (STORM; [71]) was performed on a Nikon TI-E microscope using a 100X 1.49NA objective with Perfect focus piezo Z stage controller, an Agilent solid state laser bank (405, 488, 561 and 647 nm) and an iXON3 Ultra EMCCD detector controlled by NIS elements AR software. Imaging was conducted in MatTek dishes in a buffer supplemented with glucose oxidase, catalase and cysteamine as described [72]. Recordings of 20,000 to 30,000 images, typically 500,000 to 2 million molecules, were reconstructed using parameters to optimize signal-to-noise and were corrected for drift. Inspection of molecular lists generated by image analysis revealed that individual localized molecules typically represented observations of 1200–1800 photons each with a lateral accuracy of 12–15 nm. Images were routinely filtered to require a minimum density of 10 molecules in a 70-nm radius to remove most background signal.

### Transmission electron microscopy and immuno-EM

A549 cells were grown in DMEM on Aclar plastic membranes (EM Sciences) and processed by fixation with either 3% paraformadehyde/0.05% glutaraldehyde [70] for immuno-EM or 2% glutaraldehyde for other applications. Samples for immuno-EM were prestained with antibodies directed against DNA and secondary anti-mouse Fluoronanogold Fab fragment antibodies with 1.4 nm gold and Alexafluor 488 modifications (Nanoprobes). Parallel preparations of fluoronanogold-labeled specimens were also examined using SIM to confirm nucleoid labeling. Pre-embedding silver enhancement was done as described [73] using HQ silver enhancement according to the manufacturer (Nanoprobes). Subsequently samples were washed 3x in PBS, contrast stained with 0.01% OsO_4_ in 0.1 M PBS for 15 min and dehydrated in ethanol in step wise fashion. These dehydrated Aclar membranes were then incubated in Durcopan (Sigma Aldrich) resin in a step wise gradient fashion until reaching 100% resin and then the cell containing surface of the resin was sandwiched with a second piece of Aclar. Sheets were dried in a 60 ⁰C oven, separated and thin sectioned. Images were collected with an FEI BioTwinG2 TEM equipped with an AMT XR-60 CCD detector.

### Automated image analysis of swollen mitochondria population incidence

Images from cells expressing MLS-EGFP captured using the Leica-SP8 system were processed using a “watershed” analysis to generate measures of mito-bulb incidence as follows. Multiple images were acquired for each condition. Images were collected at 63x, 2.5x zoom, at either 512 px^2^ (S1 Fig) or 2k px^2^ (Figs 8 and 9) resolution depending on the experiment, with 4x line averaging on the Leica SP8 confocal microscope. Images of cells under each desired condition were processed by FIJI. For high resolution images (2k px^2^) light application of a native FIJI smoothing function was performed prior to watershed-algorithm application to improve precision. The number of cells was counted in each image manually, using the native cell-counting tool. Images were processed using ImageJ/FIJI (https://imagej.net/Fiji/) using the “Interactive Watershed” algorithm (https://imagej.net/Interactive_Watershed) in order to separate and define individual mitochondria whose signals may appear to overlap or touch. The watershed tool isolates individual bloated mitochondrial objects, even in many cases when they overlap or touch structures above or below the focal plane. The watershed plugin was then used to export a “binary region mask” where individual mitochondrial structures are identified as separate elements, enumerated, highlighted, and counted based on qualifiers of total *area* and *circularity* parameters via FIJI’s native “Analyze Particles” tool. Resulting counts of mitochondria fitting criteria indicative of mito-bulbs were tabulated and the values normalized by the total number of corresponding cells in that image. Results are reported with a variety of size thresholds to document that the trends in the data hold at a variety of threshold settings.

### Mitochondrial RNA and DNA synthesis

Mitochondrial transcription was assessed by labeling cells for 45 min with 2 mM bromouridine prior to processing for microscopy and detection with anti-BrdU antibodies as described above. Replication was detected by labeling cells with 50 μM ethynyl-deoxyuridine (Thermo Fisher) for either 60 min (DMEM adapted A549) or 150 min (RPMI adapted MLS-mCherry expressing A549) prior to fixation. Click chemistry to couple the ethynyl group to fluorescent reporters used reagents from Thermo Fisher kits as noted in the table and followed manufacturer’s protocols. In some cases the Click reaction was allowed to proceed for 60 min. Cells were subsequently washed, immunostained with the desired antibody reagents and imaged using SIM as described above.

### Mitochondrial reactive oxygen species

#### MitoSOX

RPMI-adapted MLS-EGFP expressing A549 cells were seeded at 25,000/dish in a total of 2 ml phenol-free RPMI and grown for 4 days. Cells were loaded with Mito-SOX (in phenol-free RPMI) with a final concentration of 1.25 μM and incubated for at least 30 min prior to being subjected to live confocal imaging. For MitoSOX, specimens were scanned line by line at 600Hz, without line averaging, at a resolution of 512 px^2^ and included implementation of 488 and 514 notch filters to respective channels to prevent excitation light bleed-through.

#### roGFP2

RPMI-adapted A549 cells with a doxycycline inducible MLS-roGFP2 redox sensor were cultured in phenol-free RPMI for 4 days. 100 to 150 ng/ml doxycycline was added overnight (approximately 16 hours) prior to imaging. Images were collected via live confocal microscopy with quantitative images captured in photon-counting mode as described [42]. Excitation at 405 or 488 nm was used to induce fluorescence of the oxidized or reduced forms of roGFP2, respectively, with emission collection equally centered at 525 nm. Resulting images from each channel were divided by one another using MATLAB and presented in a JET map color scheme to show relative oxidized:reduced ratios of the fluorescent protein distribution in each imaged cell. In both cases (MitoSOX and roGFP2), live confocal imaging was conducted in photon counting mode with slight alterations to standard operation.

### Measurements of membrane potential

A549 cells expressing MLS-EGFP were preloaded for 60 min with 10 nM TMRM. Cells were subsequently imaged via live confocal microscopy as described above. Only cells depicting clear bloated and clear normal-morphology mitochondria in the same cell were imaged. Images were obtained in photon counting mode and subsequently processed in Leica’s LAS-X quantifications suite. In brief, 5 μm region-of-interest traces were carefully positioned to bisect either clearly bloated mitochondria or neighboring tubular mitochondria within the same cell. A total of 132 independent traces through mitochondria (60 bloated, 72 normal) were collected from 24 cells from 3 experiments. The ratios of TMRM:EGFP in swollen mito-bulbs were compared to that of tubular mitochondria within the same cells. Control “background” traces in vacant areas adjacent to sampled cells were made to permit background subtraction for normalization purposes. Signal was quantified from both the TMRM and EGFP channels by integrating areas under the curve from each trace. A Python program was written to extract the area under the curve for each trace between left and right values at half maximum of the peak. This data was subsequently extracted, adjusted for background, normalized and quantified in Excel. Pair wise comparison of the averaged values of the bloated and tubular mitochondria were weighed by the number of traces collected within each cell. During methods development, FCCP (Sigma) administration was used to confirm that TMRM signal derived from EGFP labeled mitochondria was representative of mitochondrial membrane potential. FCCP was injected, and mixed into imaging media, at increasing final concentrations in increments of 5 μM, every 10–20 min, until notable depolarization occurred at 15–20 μM.

## Supporting information

S1 Fig A549 cells grown in RPMI feature tubular mitochondria and fewer mito-bulbs per cell than DMEM-adapted cells. **A.** DMEM-adapted MLS-EGFP A549 cells frequently feature short nearly-spherical mitochondria termed mito-bulbs when they exceed a certain size. Scale bar is 5 μm. Leica SP8, 63x, 2.5x zoom, 512x512. The right panel shows the binarized mask of image A highlighting mito-bulbs with cross-sectional areas above 1.5 μm^2^. **B.** RPMI-adapted MLS-EGFP A549 cells feature long tubular mitochondrial networks and contain fewer mito-bulbs. Scale bar is 5 μm. Leica SP8, 63x, 2.5x zoom, 512x512. The right panel shows the binarized mask of image B highlighting mito-bulbs with cross-sectional areas above 1.5 μm^2^. **C.** A549 cells grow at similar rates in DMEM and RPMI. **D.** Histogram showing the average number (+/- standard deviation) of mito-bulbs per A549 cell grown in either DMEM or RPMI for 2 days on the imaging dish. 24 images, 130 cells. **E.** Histogram showing the average number (+/- standard deviation) of mito-bulbs per A549 cell grown in either DMEM or RPMI for 4 days on the imaging dish. 28 images, 212 cells.
(TIF)

S2 Fig SIM images depict mitochondrial inner-membrane and nucleoid arrangement in A549 cells in RPMI without and with supplemental glutamine. **A.** Normal morphology, tubular mitochondria show high density staining for the inner membrane marker (PHB2) throughout their tubular structures. SIM images as a maximum intensity projection of 7 Z-sections (total 0.875 μm) of an RPMI-adapted A549 cell expressing MLS-mCherry (blue). Cells were immunostained for Tom20 (red) and PHB2 (green). The indicated ROI in A is enlarged with channels resolved on the right. Scale bar is 2.5 μm. **B.** SIM image of a region of an A549 cell expressing MLS-EGFP (blue) grown in RPMI with 8 mM glutamine. Outer membrane marker Tom20 (green), MLS-EGFP (blue) and anti-DNA antibody stained individual and clustered nucleoids (red) are shown in the main panel B and as individual channels on the right.
(TIF)

S3 Fig Quantitative representation of a mitochondrially targeted ROS sensor show that swollen mitochondria commonly contain a higher ratio of oxidized:reduced roGFP. **A-D.** Sample images of RPMI adapted A549 cells expressing MLS-roGFP2, clearly featuring prominent mito-bulbs and normal morphology mitochondria within the same cell, imaged via confocal microscopy operating in photon counting mode and then displayed as Oxidized:Reduced channel ratios via MATLAB.
(TIF)

S1 Video (ZIP)

S2 Video (ZIP)
