# Supplementary figures and images for: A549 cells contain enlarged mitochondria with independently functional clustered mtDNA nucleoids
Source: PLoS One. 2021 Mar 25;16(3):e0249047. doi: 10.1371/journal.pone.0249047 (PMC7993880; doi:10.1371/journal.pone.0249047)

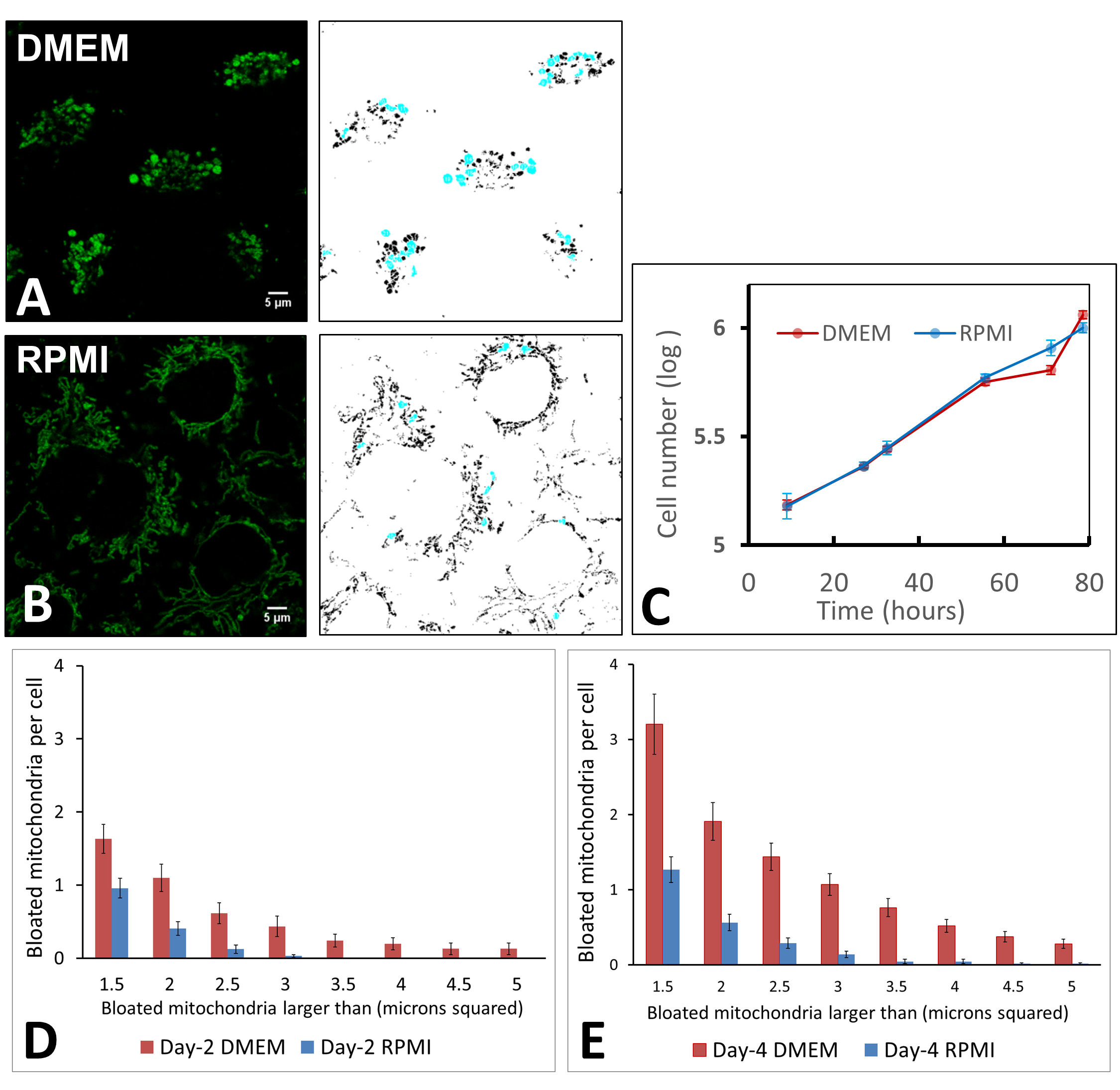

Supplement: S1 Fig — A. DMEM-adapted MLS-EGFP A549 cells frequently feature short nearly-spherical mitochondria termed mito-bulbs when they exceed a certain size. Scale bar is 5 μm. Leica SP8, 63x, 2.5x zoom, 512x512. The right panel shows the binarized mask of image A highlighting mito-bulbs with cross-sectional areas above 1.5 μm2. B. RPMI-adapted MLS-EGFP A549 cells feature long tubular mitochondrial networks and contain fewer mito-bulbs. Scale bar is 5 μm. Leica SP8, 63x, 2.5x zoom, 512x512. The right panel shows the binarized mask of image B highlighting mito-bulbs with cross-sectional areas above 1.5 μm2. C. A549 cells grow at similar rates in DMEM and RPMI. D. Histogram showing the average number (+/- standard deviation) of mito-bulbs per A549 cell grown in either DMEM or RPMI for 2 days on the imaging dish. 24 images, 130 cells. E. Histogram showing the average number (+/- standard deviation) of mito-bulbs per A549 cell grown in either DMEM or RPMI for 4 days on the imaging dish. 28 images, 212 cells. (TIF) [file pone.0249047.s001.tif]

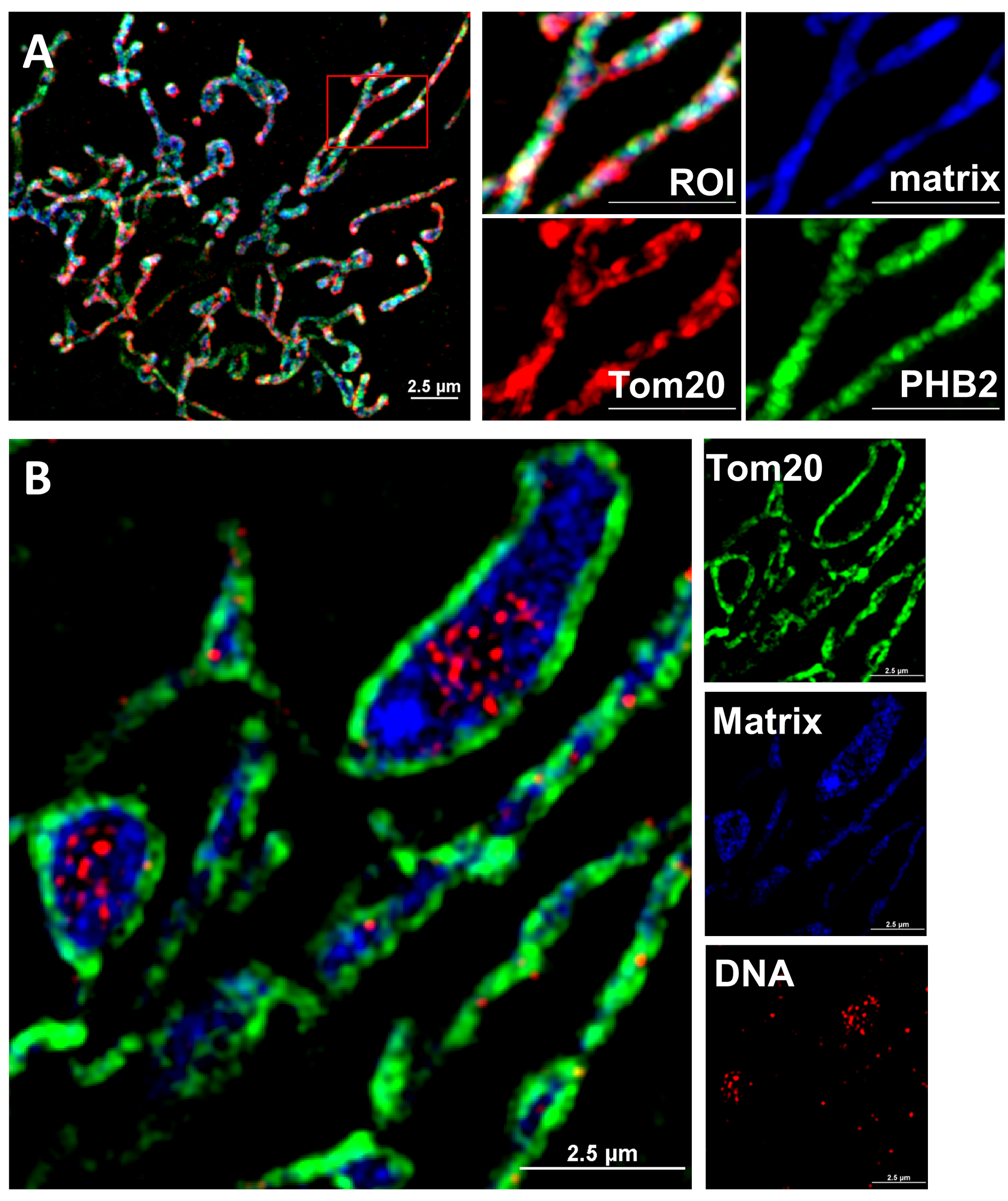

Supplement: S2 Fig — A. Normal morphology, tubular mitochondria show high density staining for the inner membrane marker (PHB2) throughout their tubular structures. SIM images as a maximum intensity projection of 7 Z-sections (total 0.875 μm) of an RPMI-adapted A549 cell expressing MLS-mCherry (blue). Cells were immunostained for Tom20 (red) and PHB2 (green). The indicated ROI in A is enlarged with channels resolved on the right. Scale bar is 2.5 μm. B. SIM image of a region of an A549 cell expressing MLS-EGFP (blue) grown in RPMI with 8 mM glutamine. Outer membrane marker Tom20 (green), MLS-EGFP (blue) and anti-DNA antibody stained individual and clustered nucleoids (red) are shown in the main panel B and as individual channels on the right. (TIF) [file pone.0249047.s002.tif]

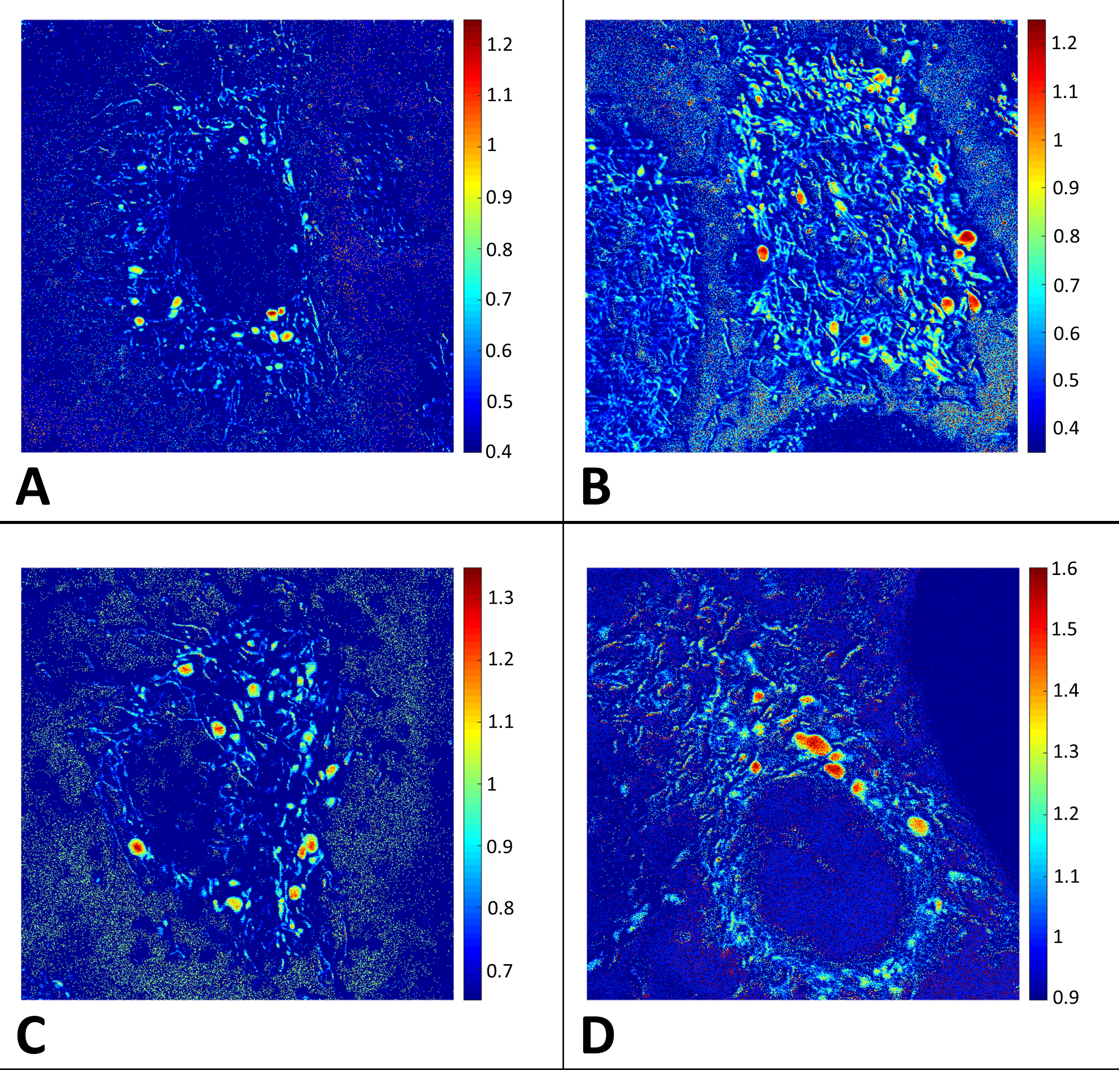

Supplement: S3 Fig — A-D. Sample images of RPMI adapted A549 cells expressing MLS-roGFP2, clearly featuring prominent mito-bulbs and normal morphology mitochondria within the same cell, imaged via confocal microscopy operating in photon counting mode and then displayed as Oxidized:Reduced channel ratios via MATLAB. (TIF) [file pone.0249047.s003.tif]
